# The Role of Tumor-Infiltrating B Cells in Tumor Immunity

**DOI:** 10.1155/2019/2592419

**Published:** 2019-09-24

**Authors:** Fei Fei Guo, Jiu Wei Cui

**Affiliations:** Cancer Center, The First Hospital of Jilin University, Changchun 130021, China

## Abstract

Earlier studies on elucidating the role of lymphocytes in tumor immunity predominantly focused on T cells. However, the role of B cells in tumor immunity has increasingly received better attention in recent studies. The B cells that infiltrate tumor tissues are called tumor-infiltrating B cells (TIBs). It is found that TIBs play a multifaceted dual role in regulating tumor immunity rather than just tumor inhibition or promotion. In this article, latest research advances focusing on the relationship between TIBs and tumor complexity are reviewed, and light is shed on some novel ideas for exploiting TIBs as a possible tumor biomarker and potential therapeutic target against tumors.

## 1. Introduction

**Figure 1:**
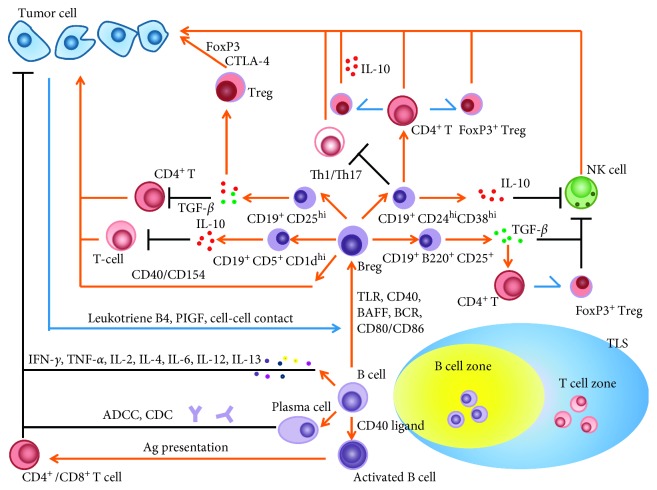
The subtypes of TIB and its dual effects on tumor. The differentiation of TIB subtypes and tumor tissue infiltration of TIB are regulated by tumor microenvironment. TIB can not only inhibit tumor development by secreting cytokines, antigen presentation, and secreting antibodies but also promote tumor progress by differentiation into Bregs, acting on tumor cells or inhibiting the function of NK and T cells and promoting the transformation of Tregs. The arrows represent the promotion effects, and the blunt ends represent the inhibition effects. The unilateral arrows represent transformation. The vertical intersection of multiple lines in the same line indicates that it points to the end of the same arrow or the end of the same blunt end.

B cells are the main humoral immune cells derived from hematopoietic stem cells that play an important role in body and antitumor immunity [1]. Nevertheless, T cells have always been considered to be the major participants in antitumor immunity, with more T lymphocytes being present in the vicinity of tumor tissues. The research on tumor immunity has also been concentrated on T cells, while the important role of B cells in this area of research has been overlooked [2]. With recent discovery of the complex correlation between B cells and tumors, especially with the discovery of tumor-infiltrating B cell (TIB) and its subtypes, and tertiary lymphoid structure (TLS), there has been an upsurge in research on B cells associated with tumors. B cells infiltrating the tumor tissues are called TIBs. Studies have shown that TIBs can differentiate into different subtypes under the influence of many factors in the tumor microenvironment, while different TIB subtypes play a dual role in tumor immunity by secreting antibodies, presenting antigens, and secreting a variety of cytokines [3]. Regulatory B cells (Bregs) belong to a subgroup of TIBs that are closely related to tumor immunosuppression. Not only do TIBs directly act on tumor cells, but they also indirectly regulate tumor immunity by affecting the function of other immune cells such as CD4^+^ T cells and Tregs and NK cells in tumor microenvironment. In addition, recent studies have described tertiary lymphoid structure (TLS), a new lymphoid tissue that contains many important immune cells such as T cells, B cells, and dendritic cells (DC). TLS is closely associated with the recruitment, activation, and proliferation of T cells and B cells. It has been reported that a portion of B cells infiltrating the tumor microenvironment exists in the B cell zone of TLS [4] (Figure 1). In addition, B cells significantly correlate with the prognosis of different types of tumors, such as breast tumor. In light of the existence of the dual role of TIBs in tumor immunity, B cell depletion therapy, and selective clearance of Bregs, promoting TLS formation as well as targeted regulation of TIB-linked signaling pathways may become effective means of TIB-based tumor immunotherapy. With increasing studies being published, B cells are also expected to become targets for tumor immunotherapy. Thus, B cells have opened new avenues for researchers to reunderstand tumor immunity and immunotherapy. To facilitate exploration of these exciting opportunities, this article aims to summarize the latest advances in the field of the tumor infiltrating B cells and ways to exploit this knowledge for tumor immunotherapy.

## 2. Biology and Characteristics of TIB

### 2.1. Recruitment of TIB

Tertiary lymphoid structure (TLS) is a recently described novel lymphoid tissue found in autoimmune diseases, chronic inflammatory diseases, and graft rejection. Under certain pathological conditions, reactivated stromal cells in local tissues provide matrix signals, induce tissue-specific overexpression of inflammatory mediators such as chemokines CCL21 and CXCL13, and recruit leukocytes to the local lesions, that together contribute to the formation of TLS. Thus, TLS is a lymphoid structure formed by ectopic aggregation of lymphocytes in nonlymphoid organs. In addition, it is a kind of ectopic lymphoid tissue which is different from the secondary lymphoid organ (SLO) in certain pathological processes that contains B cells, dendritic cells (DC), CD4^+^ T cells, CD8^+^ T cells, and other immune cells [5]. It has demonstrated that TLS can recruit, activate, and promote the proliferation of T cells and B cells and, therefore, is an important source of tumor-infiltrating lymphocytes (TIL). Recent studies have shown that TLS promotes recruitment of lymphocytes to the tumor microenvironment by expressing chemokines such as CXCL10, CXCL12, CXCL13, CCL19, and CCL21 [6]. Furthermore, it has been found that, in the presence of transforming growth factor-*β* (TGF-*β*), CD8^+^ T cells in peripheral blood can promote the recruitment of B cells to tumor tissues by upregulating the expression of CD103 and secreting CXCL13. However, inhibiting TGF-*β* receptor signal abrogates the production of CXCL13 and slows down the recruitment of B cells. Thus, it is suggested that the activation of CXCL13^+^CD103^+^CD8^+^ T cells is closely related to B cell migration and TLS formation [7].

### 2.2. Differentiation and Regulation of TIB Subtypes

Under the activation of B cell antigen receptor (BCR) pathway [8], microRNA pathway [9], and Toll-like receptor (TLR) pathway [10], B cells can proliferate, exchange phenotypes, and differentiate into effector B cells and plasma cells. Studies using the mouse model have shown that B cells are categorized into B-1a cells (B220^lower^CD5^+^), B-1b cells (B220^higher^CD5^–^), and B-2 (B220^lower^CD5^–^CD11b^+^) cells, according to their different localization and functions, in which B-2 cells are B cells in the traditional sense. In addition, B-2 cells can be divided into immature transitional cells (T1, T2, and T3) and mature follicular B cells (FOB) or marginal zone B cell (MZB). MZB cells finally differentiate into plasma cells while FOB cells differentiate into short-lived plasma cells within 1-2 and 3–5 days, respectively [11]. In addition, current studies have shown that effector B cells come from FOB cells. Furthermore, latest studies have found that long-lived follicular B cells (FOII B cells) are more likely to produce B-effector related cytokines, such as IFNG, IL-12, IL-4, and IL-2, suggesting that FOB cells have specific subsets and can differentiate into Be-1 or Be-2 cells that produce cytokines [12]. Among the subtypes of TIBs, Bregs are quite an exceptional class that has many phenotypes such as CD5^+^ Bregs, CD19^+^CD5^+^CD1d^hi^ Bregs, CD19^+^CD25^hi^ Bregs, CD19^+^CD24^hi^CD38^hi^ Bregs, and CD19^+^B220^+^CD25^+^ Bregs.

The differentiation of TIBs is regulated by various factors in the tumor microenvironment, such as the tumor type, the location and activation of B cells, and other immune cells in the tumor microenvironment. For instance, in non-small-cell lung cancer, prostate cancer, and ovarian cancer, the differentiation of CD5^+^ B cells is associated with the phosphorylation of STAT3 in tissues [13]. In breast cancer, ovarian cancer, cervical cancer, colorectal cancer, and prostate cancer, CD4^+^ T cells lacking CD40L induce B cells to differentiate into granular B-secretory cytotoxic cells by producing IL-21 [14]. Moreover, under the influence of CD4^+^ Th1 and Th2 cells in tumor microenvironment, TIBs polarize into Be-1 cells that produce IFN-*γ*, IL-12, and TNF-*α* or Be-2 cells that produce IL-2, IL-4, TNF-*α*, and IL-6 [15]. In addition, by activating the CD40 pathway or presenting IL-15 to B cells through cell-cell contact, macrophages and monocytes in the tumor microenvironment can promote the proliferation and differentiation of Bregs [16].

## 3. Tumor Inhibition by TIB

It is well known that B cells play a crucial antitumor role through a variety of ways, such as producing antibodies, forming antigen-antibody complexes, acting as antigen presenting cells (APCs), and secreting cytokines. Thus, TIBs have the potential to eradicate tumor cells or affect the function of other immune cells in the tumor microenvironment and thus achieve antitumor effect [17].

### 3.1. TIB-Derived Antibodies

It is well known that the secretion of antibodies is an important mode for B cells to exert immune effect. Studies have revealed that B cells play an antitumor role through the neutralization of antibodies, conditioning of antibodies, and the formation of antigen-antibody complexes. For instance, it has been found that TIB activated by MCA205 tumor cells produce IgG2b antibody which in turn binds to MCA205 tumor cells and thus plays an antitumor role. This binding of IgG2b antibody to MCA205 tumor cells is specific implying that the IgG2b antibody cannot bind to other tumor cells [2]. In addition, studies have shown that TIBs directly eradicate tumor cells wrapped in IgG through antibody-dependent cell-mediated cytotoxicity (ADCC) or dissolve tumor cells by antibody-mediated activation of the complement system and thus achieve antitumor immunity [18].

### 3.2. Ag Presentation by TIBs

B cell itself is a kind of antigen-presenting cell. Although inactive B cells have little antigen presenting potential, activated B cells have very dominant antigen-presenting functions. Studies have shown that the costimulatory molecules, chemokines, and adhesion molecules on the surface of B cells are highly expressed under the action of antigen receptor, lipopolysaccharide, and CD40 ligand, thus greatly enhancing the antigen presentation ability of B cells. For example, B cells activated by CD40 ligands highly express chemokine and costimulatory factors such as CCL2, CXCR4, CCL5, CXCL5, and CXCL10 and induce antigen-specific CD8^+^ CTL cell and CD4^+^ T cell reaction to exert antitumor immune effect [19]. Compared with that by DC, a traditional antigen-presenting cell, TIBs present antigen in the tumor microenvironment under the stimulation of low-concentration antigen, and the process of antigen presentation lasts longer. TIBs have the potential to function as local APCs in tumor tissue and maintain the survival and proliferation of tumor infiltrating T cells for a long time, thus assisting antitumor immunity [20].

### 3.3. Cytokine Secretion by TIBs

B cells secrete a variety of cytokines to regulate tumor immunity while themselves being heterogeneous in cytokine production. For example, Be-1 cells initiated by Th1 cells and antigens produce cytokines like IFN-*γ*, TNF-*α*, and IL-12, while Be-2 cells initiated by Th2 cells and antigens produce cytokines such as IL-2, IL-13, TNF-*α*, IL-6, and IL-4 [21]. These cytokines play significant roles in antitumor immunity. Studies have shown that IL-12, a heterodimer cytokine produced by B cells, induces the production of various cytokines such as IFN-1 and promotes the proliferation and antitumor effects of T and NK cells. Moreover, the gene therapy scheme for IL-12 has been formally proposed implicating its crucial contribution towards antitumor immunity [22].

Through the abovementioned ways, TIB can interact with tumor cells and other immune cells in tumor microenvironment and play an antitumor effect. Among them, the interaction between B cells and T cells is considered to be the key to activate local immune response under a variety of inflammatory conditions, including cancer. Garnelo et al. found that, in hepatocellular carcinoma (HCC), tumor-infiltrated T cells and B cells were in close contact with each other and formed tertiary lymphoid structure. In addition, the density of TIB was related to the enhanced expression of granzyme B and IFN-1, both of which are the activation markers of cytotoxicity T and NK cells. Therefore, the density of TIB was closely related to the activation of CD8^+^ T and CD56^+^ NK cells in tumor microenvironment, which in turn may lead to the enhancement of local antitumor immune response and suggest a good prognosis [23]. In addition, Dieu-Nosjean et al. found that there were tertiary lymphoid structures composed of mature DC/T cell clusters and B cell follicles in non-small-cell lung cancer (NSCLC), in which the B cell area included proliferating GC B cells and follicular DC networks, and persistent immune responses were observed in typical lymphoid organs, suggesting that proliferating GC B cells may be active partners in local initiation of potential antitumor immunity [24]. In addition, Nielsen et al. found that, in high-grade serous ovarian tumors, CD20^+^ TIL colocated with activated CD8^+^ TIL and expressed antigen presentation markers, including MHC class I, MHC class II, CD40, CD80, and CD86, and the presence of both CD20^+^ and CD8^+^ TIL correlated with increased patient survival compared with CD8^+^ TIL alone [25]. However, although studies on B cells and T cells continue to emerge, more research is needed on the mechanism of interaction between B cells and T cells in different tumor types.

## 4. TIB-Mediated Tumor Promotion

Apart from positive regulation of antitumor immune process, TIB can also negatively regulate antitumor immune response. This process is mainly attributed to the function of Bregs, a special subgroup of B cells. Toll-like receptor pathway (TLR), CD40 pathway, B cell activating factor pathway (BAFF), B cell receptor pathway (BCR), and CD80/CD86 are closely related to the differentiation of Bregs [26]. Moreover, tumor cells can induce the formation of Bregs through cell-cell contact or release of soluble factors such as leukotriene B4 and glioma-derived placental growth factor [27]. Reports have shown that activated Bregs negatively regulate antitumor immunity by affecting the function of other immune cells in addition to promoting the occurrence and development of tumor by acting on tumor cells.

### 4.1. Effect of Bregs on Tumor Cells

For TIBs, few studies have shown that B cells produce lymphotoxin and promote the progress of androgen-resistant prostate cancer by activating the IKK*α*-BMI1 signal pathway in prostate cancer stem cells under the influence of chemokine CXCL13 [28]. For Bregs, granzyme B expressing Bregs, induced by tumor cells and IL-21, react to tumor cells and promote tumor immune escape. It has been reported that Bregs secreting transforming growth factor-*β* (TGF- *β*) affect the epithelial-mesenchymal transition (EMT) in tumor tissues [29]. For instance, studies have confirmed the synergistic effects of TGF-*β* produced by Bregs in association with Ras and Wnt signaling pathways in epithelial cell carcinoma [30] and colorectal cancer [31], respectively, that induces EMT and promotes the process of disease to a certain extent. In addition, it has been found that, in patients with primary liver cancer (HCC), Bregs directly interact with cancer cells through CD40/CD154 signaling pathway and promote the growth and infiltration of HCC cells [32].

### 4.2. Effect of Bregs on Other Immune Cells

In addition to acting on tumor cells, Bregs exhibit a tumor promoting effect by acting on other immune cells in the tumor microenvironment. It has been shown that Bregs negatively regulate the antitumor immune function of T cells by secreting cytokines such as IL-10, TGF-*β*, and IL-35. Among various phenotypes of Bregs, CD19^+^CD5^+^CD1d^hi^ Bregs, also called B10 cells, secrete IL-10 and promote the transformation of Th0 to Th1 and Th2, thereby inhibiting the proliferation and activation of T cells [33]. Besides, CD19^+^CD25^hi^ Bregs inhibit the proliferation of CD4^+^ T cells by secreting IL-10 and TGF-*β* and promote the expression of FoxP3 and CTLA-4 in the Tregs, both of which are Tregs tumor suppressor markers [34]. CD19^+^CD24^hi^CD38^hi^ Bregs not only inhibit the proliferation of CD4^+^ T cells and the differentiation of Th1/ Th17 but also promote the transformation of CD4^+^ T cells to FoxP3^+^ Tregs and the Tregs secreting IL-10 [35]. Previous studies have shown that IL-35 is mainly produced by Tregs and inhibits the proliferation and immunoregulation of T cells in vitro, but recent studies have found that Bregs are also an important source of IL-35. Moreover, the loss of IL-35 leads to the increase in the activation of macrophages and T cells as well as the increase in the B-cell antigen-presenting ability [36]. In addition, it was found that Bregs induce T cells to lose function and apoptosis by cell-cell contact, thus inhibiting the proliferation of T cells [37].

In addition to acting on T cells, Bregs promote tumor by acting on other immune cells such as NK. For example, studies have shown that, in myeloma patients, CD19^+^CD24^hi^CD38^hi^ Bregs can eliminate the toxic effect of NK cells on myeloma cells by secreting IL-10 [38]. In the mouse breast cancer model, CD19^+^B220^+^CD25^+^ B cells inhibit T cell production by producing TGF-*β* and contact with cells and induce the transformation of young CD4^+^ T cells to FoxP3^+^ Tregs. The transformed Tregs further inhibit the antitumor effect of NK cells and promote tumor metastasis [39]. Apart from that, STAT3 is a favorable transcription factor for IL-10. It has been found that CD5^+^ Bregs inhibit tumor immune response by activating STAT3, thus promoting tumor development [13].

## 5. TIBs and Prognosis of Cancer Patients

Many studies have revealed a significant correlation between tumor infiltrating lymphocytes (TILs) and clinical prognosis in cancer patients. TIL has also been identified as a prognostic and predictive biomarker for several cancers, including breast cancer [40]. Recently, in addition to T cells, TIBs have gradually attracted the attention of researchers (Table 1). Various studies have shown that TIBs are related to better prognosis of cancer patients including the liver, colorectal, bladder, breast, lung, and ovarian cancer [25].

The distribution, phenotype, and function of CD20^+^ TIBs in HCC have been analyzed by Shi et al. It was found that CD20^+^ TIBs mainly infiltrate at the edge of the tumor compared with the peri- and intratumoral areas and form a dense cell layer in the invasive marginal area which contains a large number of CD8^+^ T cells. The results indicated that CD20^+^ TIBs are positively correlated with small tumor size, absence of vascular invasion, and increased density of CD8^+^ T cells. The survival rate analysis showed that the increase of CD20^+^ TIBs, its penetration through tumor capsule, and its synergistic effect with CD8^+^ T cells are significantly correlated with the overall and recurrence-free survival rate of HCC. The CD20^+^ TIBs have also been identified as an independent prognostic index for patients with hepatocellular carcinoma [41]. It has been found that, in colorectal cancer, the high density of CD20^+^ TIL and the expression of specific CD138^+^ and immunoglobulin kappaC (IGKC) in immune cells were significantly correlated with the improvement of OS [42]. In addition, in muscle-invasive bladder cancer (MIBC), CD19^+^ TIB was identified as an independent prognostic factor and serve as antigen-presenting cells (APCs) to activate CD4^+^ T cell in the tumor microenvironment; the OS of the high CD19^+^ TIB MIBC patient was significantly longer [43]. Moreover, a study found that, in inflammatory breast cancer (IBC) and triple-negative inflammatory breast cancer (TNIBC), CD20^+^ TIL/PD-L1^+^ TIL is an independent prognostic factor for IBC and TNIBC, suggesting that B cells play an important role in antitumor immune response and anti-PD-1/PD-L1 and B-cell activated immunotherapy should be further discussed [44]. In lung cancer, especially in the adenocarcinoma, the tumor-infiltrating CD138^+^ plasma cells and IGKC^+^ immune cells, but not the CD20^+^ B cells, were found to be related to the survival rate in single and multifactor analysis [45].

In addition, several latest studies have discussed the prognostic value of TLS in tumors. A study of liver cancer found that about 6% of patients with liver cancer have TLS formation, and the formation of TLS is related to the improvement of survival rate [46]. In breast cancer, about 37% of the patients are detected with TLS, and the existence of TLS is related to the nonmetastatic survival of hormone receptor negative breast cancer patients [47]. For patients with colorectal cancer [48], melanoma [49] and gastric cancer [50], the presence of TLS in or around the tumor is also a good prognostic indicator.

However, there are also few studies showing that TIBs are related to the poor prognosis of some tumors, such as prostate cancer [51], breast cancer, ovarian cancer [52], lung cancer [53] and melanoma [54]. It has been found that Higher CD20^+^ TIB is related to high grade ductal carcinoma in situ (DCIS). As research on the role of TIB in the preinvasive phase of breast cancer becomes clearer, it may provide new options for preventing breast cancer from developing [55]. In addition to good prognosis and poor prognosis, some studies have confirmed that TIB is not related to the prognosis of some tumors, such as node-negative breast cancer [56] and NSCLC [57]. Thus, the mechanism of different effects of TIBs on the prognosis of various tumor tissue types needs to be further determined [58].

## 6. Therapeutic Application of TIBs

TIBs play a dual regulatory role in tumor immunity. Thus, the effective usage of TIBs to enhance antitumor immunity is very vital. The potential mechanisms and strategies for the application of TIB in tumor therapy have been continuously investigated and proposed. Many studies have shown that B-cell-depletion therapy and selective clearance of Bregs, the TLS formation, immune checkpoint, and other targeted regulation of TIB-related signaling pathways have the potential to be explored as effective approaches for tumor immunotherapy. Moreover, combinatorial strategies such as B-cell and T-cell epitopes [59] may also be an effective treatment. With the deepening of TIB research, TIB may bring new dawn to the clinical treatment of tumor patients.

Promoting tumor immunity by depleting immunosuppressive B cells, i.e., B cell depletion therapy (BCDT), has shown some positive antitumor effects. This is because tumor infiltration of immune cells such as Bregs and Tregs can induce immune tolerance and some positive anticancer effects have been shown by depleting immunosuppressive B cells to weaken immune tolerance. For example, it has been found that the depletion of B cells enhances the recruitment of CD4^+^ T cells and CD8^+^ T cells to tumor microenvironment, thereby providing explanation for the tumor model of mice with B cell deficiency being very sensitive to chemotherapy and more effective against tumor immunity [60]. It was also found that the number and function of CD4^+^/CD25^+^/FoxP3^+^ Tregs in the lymph nodes, spleen, and tumor tissue are reduced in the B-cell-deficient mouse model, resulting in significant inhibition of tumor development [61]. Although the B-cell-depleted antibody is a commonly used cytotoxic drug in human B-cell-related malignancies, it is rare to use B cells as a means to regulate the immune response of a non-B-cell tumor. However, more and more studies have shown that B cell depletion with anti-CD20 antibody can inhibit tumor growth and effectively enhance immunotherapy in a variety of solid tumor models. This suggests that BCDT may be a useful auxiliary in human immunotherapy [62].

However, although the effectiveness of BCDT has been verified to some extent, the specificity of the markers used to distinguish Bregs is still not adequate for the application of Bregs in cell therapy. That is to say, using anti-CD 20 antibody, there is no way to specifically consume mature B cells with antitumor effect or Bregs with tumor-promoting effect. This is because the current clearance of B cell antibodies does not effectively distinguish effector B cells from Bregs. Thus, identification of the specific targets of Bregs subtypes and the approaches for their selective elimination need to be further explored. Through the removal of TIB with specific markers, such as the subtypes of Bregs, CD19^+^CD5^+^CD1d^hi^ Bregs, CD19^+^CD25^hi^ Bregs, CD19^+^CD24^hi^CD38^hi^ Bregs, and CD19^+^B220^+^CD25^+^ B cells, it is possible to provide more effective therapeutic effect for BCDT.

Recent studies have revealed the beneficial effects of TLS on the survival rate of tumor patients, thus providing a new avenue in terms of TLS formation in tumor microenvironment for improving the efficiency of immunotherapy. A breast cancer study has shown that patients with hormone receptor-negative breast cancer have a long disease-free survival time (DFS) in the presence of TLS, but the same is not true in case of hormone receptor-positive breast cancer patients [47]. In addition, it has been observed that the expression of TFH gene reflects the formation of TLS in tumor microenvironment, while the use of cytokines such as CXCL13 and lymphotoxins in tumor microenvironment also stimulates the TLS formation and promotes the recruitment of immune cells [63]. Thus, to exploit the phenomenon of TLS formation for antitumor immunotherapy, the mechanism of TLS formation and its role in different tumor types need to be further explored.

In addition, some studies have shown that the immune checkpoint also plays an important role in TIB-related tumor immunotherapy. A novel protumorigenic PD-1^hi^ B-cell subset, CD5^hi^CD24^–/+^CD27^hi/+^CD38^dim^ B cell, was found in human HCC. A study found that Toll-like receptor-4- (TLR4-) mediated proto-oncogene BCL6 upregulation is critical for induction of PD-1^hi^ B cells, which operate via IL10-dependent pathways upon interacting with PD-L1 to cause T-cell dysfunction and foster disease progression, and that effect was abolished by IL4-elicited STAT6 phosphorylation. It is possible to provide significant new insights for human cancer immunosuppression and anticancer therapies regarding PD-1/PD-L1 [64]. In lung adenocarcinoma (LUAD), PD-L1 upregulates the function of infiltrating T cells in LUAD. In addition, Ho et al. found that TIB was related to PD-L1, and it can be used as the clinical factors of anti-PD-L1 immunotherapeutic LUAD [65]. In addition, Das et al. found that checkpoint blockade may impact B cell function by directly acting on B cells expressing the specific checkpoint or indirectly via effects on T cells or myeloid cells, and it may be related to immune-related adverse events (IRAEs) [66]. This indicates that there is a correlation between TIB and immune checkpoint and immune checkpoint inhibitors. The study of immune checkpoint and TIB may provide new opportunities for immunotherapy of different types of tumors.

In addition to the above pathways, accurate targeted therapy for TIB-related signaling pathways may also be a potentially effective treatment, including signaling pathways and molecules related to TIB development and differentiation, such as the CD40/CD40L pathway, the BAFF/BAFFR pathway, B-cell translocation gene 1, TNF family, and microRNAs.

CD40 is an important B cell surface molecule mediating T cell response. B-cell-activating factor (BAFF) is a member of tumor necrosis factor (TNF) family, which promotes the proliferation, maturation, and immunoglobulin secretion of B cells. The CD40/CD40L pathway, the BAFF/BAFFR pathway, is closely related to TIB; CD40 stimulation has produced impressive results in early clinical trials of cancer patients and a better understanding of how CD40 receptors are activated, and the subsequent function of CD40 stimulating immune cells will contribute to further progress. Thus, stimulating the CD40/CD40L pathway can be explored as an effective antitumor strategy. For instance, after stimulation of CD40, B cells can replace DCs as APCs to activate T cells. As B cells proliferate more easily than DC in vitro, they provide new avenues with great potential to be explored for adoptive cellular immunotherapy [67].

In addition, TNF family and its downstream signaling pathway, B-cell translocation gene 1 (BTG1), and microRNAs [68] play an important role in the regulation of B cell differentiation, proliferation, and apoptosis. Among which, drugs targeting TNFs and its ligands have achieved promising results in clinical trials [69]. In HCC, mRNA and protein levels of BTG1 were decreased and downregulated BTG1 mRNA was significantly associated with the survival rate of HCC [70]. As can be seen from the above, the research on the application of TIB in tumor immunotherapy is very rich, including many directions. However, some of the specific mechanisms are not completely clear, and there is a long way to go to apply it to the clinical treatment of tumor patients. Therefore, this field needs to be further explored.

## 7. Conclusion and Perspectives

Our previous understanding of the role of B cell in tumor immunity is its antitumor effect. However, further evidence suggests that B cell can also promote tumorigenesis by regulating immune response. Therefore, the role of B cell in tumor immunity is becoming more and more complex, which may become an important factor in tumor immunotherapy. Based on the current research on TIBs, it has been found that TIBs can differentiate into different subtypes under the regulation of many factors in tumor microenvironment. This reminds us that we can try to regulate the differentiation of special subtypes of TIB by controlling the relevant conditions, so as to play a different role. These subtypes, in turn, play a complex dual role in tumor immunity through the secretion of cytokines and antibodies and Ag presentation. In addition, the significant association between TIB and the prognosis of tumor patients has been confirmed, and many studies have shown that B-cell-depletion therapy and selective clearance of Bregs, the TLS formation, immune checkpoint, and other targeted regulation of TIB-related signaling pathways may be effective strategies for tumor immunotherapy. According to the difference of special markers on the surface of TIBs, TIBs have many different characteristic that can be defined as different subtypes. Through some experimental techniques to obtain more special phenotypes, making the relation between TIBs subtypes and tumor types more clear, it is possible to subdivide TIBs into more subgroups, and it may be an effective way to study the effect of TIBs on the prognosis of patients with different tumor types from bulk or at the single cell level. Few of the above approaches have begun to be employed in the clinics and have shown broad prospects. On the basis of the established research, how to determine the relationship between different TIB subtypes and the prognosis of patients with various tumors, how to selectively remove TIB with special phenotype in BCDT, and how to find and select more efficient specific therapeutic targets at the molecular level and apply it to clinic are supposed to be the key to TIB research in the future. Although the complete mechanisms specific to the interaction between TIBs and tumors are still not fully understood, detailed studies elaborating TIB function and its subtypes may provide a new way for cancer screening, prediction, and development of prognostic markers. Thus, TIBs have a great potential to facilitate the reunderstanding of tumor immunity and immunotherapy.

## Figures and Tables

**Table 1:** TIB and prognosis of cancer patients.

Tumor type	Related mechanism	Prognostic evaluation
Breast cancer
	TLS formation, CD20^+^ TIB, **IBC and TN IBC**: CD20^+^ TIL/PD-L1^+^ TIL	Good

	**DCIS**: CD20^+^ TIB	Bad
	**Node-negative breast cancer**	Unrelated

Ovarian cancer
	**High-grade serous ovarian tumor**: correlation of antigen-experienced but atypical CD27^–^CD20^+^ TIL and CD8^+^ T cell	Good
	CD138^high^CD20^+^ TIB	Bad

Melanoma
	TLS formation, high CD20^+^ TIB, expression of CD20^+^ TIB in close proximity to CD3^+^ T cells	Good
	**Cutaneous melanoma**: high CD20^+^ TIB, CD138^+^ plasma cells	Bad

Colorectal cancer	TLS formation, CD20^+^, CD138^+^, IGKC^+^ tumor-infiltrating B cells and plasma cells	Good

Gastric cancer	TLS formation	Good

Bladder cancer	**MIBC**: CD19^+^ TIB serve as APC to activate CD4^+^ TIT	Good

Prostate cancer	Higher TIB	Bad

Lung cancer
	**Adenocarcinoma**: CD138^+^ plasma cells, IGKC^+^	Good
	**NSCLC**: CD20^+^ B cells and CD79*α*^+^p63^+^ plasma cells	Bad
	**NSCLC**	Unrelated

Liver cancer	TLS formation	Good
	**HCC**: synergistic effect of CD20^+^ TIB and CD8^+^ T cell	

Special tumor subtypes are shown in bold.

## Data Availability

The data used to support the findings of this study are included within the article.
